# Identification of Risk of Early Decompensation and Predictors of ICU Admission in Patients Triggering Code Sepsis

**DOI:** 10.7759/cureus.77026

**Published:** 2025-01-06

**Authors:** Muhammad Saad Anwar, Poorva Bhide, Gaurav Mohan, Anosh Aslam Khan, Sai Gaddameedi, Fatima Kausar Nawaz, Hamzeh Nasr, Mahrukh A Khan, Farhan Khalid, Doantrang Du

**Affiliations:** 1 Internal Medicine, Monmouth Medical Center, Long Branch, USA; 2 Internal Medicine, The Wright Center, Scranton, USA; 3 Internal Medicine, Robert Wood Johnson Barnabas Health, Long Branch, USA

**Keywords:** criticial care, icu, qsofa, sepsis, sirs

## Abstract

Introduction

“Code Sepsis” is a protocol, auto-initiated as a system-based trigger in our community-based teaching center. It is designed to be activated in patients with two or more systemic inflammatory response syndrome (SIRS) criteria and organ dysfunction. The purpose is to identify sepsis early and treat it according to the Surviving Sepsis guidelines. If clinically indicated, patients are rapidly evaluated and treated with antibiotics and fluid boluses. Despite proactive measures to improve patient outcomes, clinical deterioration can still occur, which may necessitate ICU admission, vasopressor initiation, and even increased morbidity and mortality. In this study, we aimed to compare patients who triggered a code sepsis requiring ICU admission at a teaching hospital with those who did not require ICU admission, identifying risk factors for early decompensation.

Methods

We conducted a retrospective study to gather data on all patients admitted to the hospital between September 1, 2022, and December 31, 2022, who triggered code sepsis. Subjects were identified based on whether they triggered code sepsis, and demographic details, admission details, laboratory values, and code sepsis notes were collected using a Redcap questionnaire. The data was reviewed and evaluated using STATA software (Stata Corp., College Station, TX, USA), and means were calculated and compared between the two outcome cohorts. Multivariate logistic regression analysis was performed to determine adjusted odds ratios (OR).

Results

For patients who triggered code sepsis, the mean age (63.94 vs. 63.71, p=0.963) and mean length of stay (11.94 vs. 10.03 days, p=0.422) were comparable between those who required ICU admission and those who did not. However, there were significant differences in other factors. The mean initial lactate (2.22 vs. 1.40, p=0.017), initial alanine aminotransferase (ALT) level (74.52 vs. 37.11, p<0.05), and aspartate aminotransferase (AST) levels (149.84 vs. 61.03, p=0.005) were significantly higher in patients who required ICU level care. At the time of code sepsis, patients who needed a 30 cc/kg fluid bolus (OR=12.8, p<0.01), or had hypotension in the first hour after the event (OR=7.94, p<0.01) had a higher chance of requiring ICU admission. Patients meeting quick sequential organ failure assessment (qSOFA) criteria (OR= 4.4 and 5, p<0.01) and requiring escalation of antibiotics (OR=19.33, p<0.01) at the time of code sepsis were also more likely to require ICU. White cell count, glucose level, serum creatinine, and troponins at admission were comparable in both groups. There was no statistically significant difference in the distribution of co-morbidities such as type 2 diabetes, hypertension, cardiovascular, or kidney disease among the patients who went to the ICU versus those who did not.

Conclusion

Code sepsis, triggered by the system or physician, identifies the presence of SIRS and organ dysfunction, thus enabling healthcare providers to intervene and manage sepsis or septic shock earlier. Higher lactate levels, the presence of transaminitis during the initial trigger event, and positive qSOFA criteria indicate a worse prognosis. This may require escalation of care to the ICU, closer monitoring, and possible use of vasopressor support. Early identification of such individuals can lead to better management of their condition.

## Introduction

Surviving sepsis guidelines recognize sepsis as fatal organ dysfunction secondary to dysregulated host response in the infection setting, consistent with the definition proposed by the Third International Consensus Task Force (Sepsis-3) [1]. It has contributed to approximately 20% of global deaths, with mortality rates as high as 45% [2,3]. Early and accurate diagnosis and management of sepsis remain challenging due to the variable presentation of infectious etiology, the severity of illness, the site of infection, and the antimicrobial resistance pattern confounded with the presence of multiple comorbidities. Delayed recognition of sepsis may cause rapid clinical deterioration, leading to transfer to ICU or death, and increasing hospital length of stay, resulting in billions of dollars in healthcare costs worldwide [4,5]. To reduce such high mortality and morbidity, several guidelines and strategies have been devised to increase sepsis awareness and direct therapy [1,3]. Henceforth, the Surviving Sepsis Campaign recommends the implementation of sepsis bundles with risk stratification to identify early warning signs that use routinely available clinical data and can alert clinicians to intervene in patients at risk for impending deterioration [1]. Research has demonstrated that patients often display early signs that indicate a high risk of sepsis, such as abnormal vital signs, or laboratory results such as elevated lactic acid and serum creatinine. These indicators can predict the patient’s clinical status over the next few hours [4,6]. As a result, many institutions have implemented automated sepsis screening tools to facilitate the diagnosis and management of sepsis.

Many hospitals use automated screening tools to detect sepsis based on systemic inflammatory response syndrome (SIRS) criteria, with modifications tailored to each hospital system. In our community-based academic hospital, sepsis alerts are based on SIRS criteria with signs of organ dysfunction. SIRS criteria is based on four components namely tachycardia (heart rate >90 beats/min), tachypnea (respiratory rate >20 breaths/min), fever or hypothermia (temperature >38°C or <36°C), and leukocytosis, leukopenia, or bandemia (white blood cells >1200/mm^3^, <4000/mm^3^, or bandemia ≥10%). These alerts are 80%-100% sensitive and 60%-90% specific in detecting sepsis [7]. When patients trigger sepsis alerts, 40% of them require escalation of care. However, not all patients with sepsis alerts need to be transferred to the ICU [8]. Our study aims to identify specific objective parameters such as laboratory findings, clinical factors, response to initial intervention, and demographic and co-morbidities of the patient that can predict ICU admission. By doing so, we hope to refine multidisciplinary efforts to manage septic patients by alerting the ICU team in advance regarding potential transfer for the need for vasopressor support. Early stratification of patients with specific high-risk features will enable the ICU team to provide timely and effective care to those who need it the most.

This article was previously presented as a rapid-fire oral presentation at the CHEST conference held during October 8-11, 2023 in Honolulu, Hawaii.

## Materials and methods

Design and data source

This was a retrospective study carried out on adult patients (aged over 18) who were hospitalized in a community teaching hospital from October 1, 2022, to December 31, 2022. The study focused on patients who were present in the emergency, medical, and surgical units and were identified as having triggered a code sepsis in the hospital system. These alerts are triggered on the basis of SIRS criteria. The sepsis alerts process vitals and laboratory values every six hours and triggers if criteria are met. Once code sepsis is responded the alerts are suppressed for the next 48 hours. The study involved accessing the medical record numbers (MRNs) of all patients who had a sepsis alert during the stipulated time frame and subsequently accessing all clinical data through the hospital's electronic medical system, Cerner.

Study population

This study included all admitted patients triggering code sepsis from October 1, 2022, to December 31, 2022, and met the inclusion and exclusion criteria, which makes a total of 120 patients. The study only included patients who triggered code sepsis that was not canceled or attributed to a different clinical condition such as sickle cell crisis and cirrhosis, as these clinical conditions can have baseline hypotension and/or fulfill SIRS or qSOFA criteria without being in sepsis. Patients were excluded if they were diagnosed with a condition other than infection that contributed to the clinical triggers for the code sepsis or had an advance directive for comfort care and non-aggressive measures, including not wanting vasopressor support. Demographic details, admission information, laboratory values, and code sepsis notes were reviewed and recorded using a Redcap questionnaire.

Outcome measures

The study aimed to compare the patient characteristics and code sepsis triggering data patterns among two groups: patients who required ICU admission for vasopressor support and those who did not. We also analyzed the distribution of patient demographics and comorbidities among both groups. We identified the predictors of a more severe clinical presentation, which was represented by the need for vasopressor support. The secondary outcomes of the study included mortality and length of stay as measures of healthcare utilization cost for both groups. Additionally, we compared other parameters of hospitalization, such as the usage of the full 30 cc/kg fluid bolus, the characteristics of the intervention at the time of code sepsis, and the escalation of antibiotic use, to identify any factors that were positively associated with the incidence of ICU admission.

Statistical analysis

Data was reviewed and analyzed using Stata® (Statistics and Data) Version 17 BE software (StataCorp, Texas, USA). Means were calculated and compared between the two outcome cohorts, and multivariate logistic regression analysis was done to calculate adjusted odd ratios (OR). A t-test was used to assess the statistical difference between the means of the two groups. Multivariate regression analysis was done to adjust for possible confounders while calculating the primary and secondary outcomes. Multivariate Analysis of Variance (MANOVA) was used to assess statistically significant differences between dependent variables. The patient and hospital characteristics, as well as comorbidities, were obtained during the literature review. A univariate screen was done to confirm these factors further. Variables with p<0.2 in the univariate screen were included in the multivariate regression model. A p-value of 0.05 was set as the threshold for statistical significance in the multivariate regression analysis. The OR was calculated for all outcomes.

Ethical considerations

The hospital database used in the study did not include any patient identifiers. Instead, all the data collected from the Hospital EHR was entered through the Redcap questionnaire, which is in compliance with HIPAA regulations. This approach ensures patient privacy and anonymity. Therefore, all NIS-based studies are exempt from institutional review board approval.

## Results

A total of 120 hospitalized patients triggered code sepsis during the six-hour system-based time window. Of these, seven were excluded based on exclusion criteria. 82 patients were included in this study, with a mean age of 64. There were no statistically significant racial differences among the patients who required ICU admission. Only eight (9.7%) were uninsured, while the rest had either Medicaid (n=5; 6.09%), Medicare (n=49; 59.75%), or Private insurance (n=20; 24.3%). The average length of stay for patients was 10 days. The majority of patients who had code sepsis were distributed as follows: six from the Emergency Room, 65 from the inpatient medical service, and 11 from the inpatient surgical service.

To study the effect of different variables on the risk of ICU admission, the sample was divided into two groups: those who were admitted to the ICU (n=19; 23.17%), and those who did not need ICU management (n=63; 76.82%). Categorical variables, including age, race, insurance status, location of admission, and initial laboratory values, were compared between these two groups using the T-test. 

For the patients who triggered code sepsis, the mean age (63.94 vs. 63.71, p=0.963) and mean length of stay (11.94 vs. 10.03 days, p=0.422) of those who required ICU admission were comparable to those who did not. The mean initial lactate (2.22 vs. 1.40, p=0.017), initial alanine aminotransferase (ALT) level (74.52 vs. 37.11, p=0.039), and aspartate aminotransferase (AST) levels (149.84 vs. 61.03, p=0.005) were significantly higher in the patients that required ICU level care (Table 1).

**Table 1:** Mean values of significant parameters for patients who required and did not require ICU level care (T-test used to calculate p-value) AST: aspartate aminotransferase; ALT: alanine aminotransferase; ALP: alkaline phosphatase

Variable	Mean	Mean		
	Patients who required ICU level care	Patients who did not require ICU level care	T-value: 1.664	P-value (P <0.05)
Age	63.94	63.71	-1.81	0.963
Length of stay	11.94	10.03	0.1974	0.422
Hospital day	2.47	2.49	-2.157	0.983
Lactic acid	2.22	1.4	2.157	0.017
White blood cell count	13.42	12.36	-0.1769	0.57
Blood sugar level	122.63	136.19	0.121	0.452
Creatinine	1.17	1.04	-0.1565	0.562
Troponins	0.208	0.232	-1.563	0.939
AST	149.84	61.03	2.638	0.005
ALT	74.52	37.11	1.785	0.039
ALP	196.47	164.3	-0.1744	0.569

The data were again divided into two cohorts: those who were admitted to the ICU and those who did not require ICU management, to study the effect of the features of the code sepsis event, including the nature of the SIRS criteria that triggered the event and various forms of intervention undertaken during the management of the patients. Multivariate logistic regression analysis was performed to calculate adjusted OR as shown in Table 2.

**Table 2:** OR calculated through multivariate logistic regression analysis reflecting the higher risk of ICU admission. MANOVA was used to determine the statistical significance MANOVA: Multivariate Analysis of Variance; qSOFA: quick sequential organ failure assessment; OR: odds ratio

Variable	Reference range	Odds ratio	F-value	P-value (p <0.05)
SBP <90 or MAP <65, or SBP decrease of more than 40 mmHg	-	1.35	0.3271	0.569
Creatinine >2 or urine output <0.5 mL/kg/hr for 2 hours	0.6-1.2 mg/dL	N/A	-	N/A
Bilirubin >2	0.2-1.2 mg/dL	1.96	1.36	0.247
INR >1.5 or a PTT >60 sec	INR: 0.8-1.2, PTT: 25-35 sec	0.34	2.582	0.112
Platelets <100000	140-450 x 10^3^/μL	N/A	0.3071	0.581
Lactate >2 mmol/L	<2 mmol/L	2.45	2.071	0.154
Heart rate >90	60-100 beats/min	1.79	0.5318	0.468
Respiratory rate >20	12-20/min	3.69	4.822	0.032
Temperature >3.83°C/100.9F or <36.0°C/96.8F	97-99F (36.1-37.2°C)	0.55	0.7663	0.384
WBC >12000 or <4000 or >10% bands	4.5-11x10^3^/μL	0.87	0.04979	0.824
Administration of total 30 cc/kg IV fluid bolus	-	12.8	11.67	0.001
Hypotension postcode sepsis	-	7.94	-	0
Ongoing antibiotic therapy	-	1.07	0.01996	0.888
Ongoing broad spectrum therapy	-	2.44	1.502	0.224
Antibiotics escalation at the code sepsis	-	19.3	10.21	0.002
qSOFA assessment-respiratory rate >22	-	2.14	1.515	0.222
qSOFA assessment-SBP <100 mmHg	-	4.4	8.335	0.005
qSOFA assessment-mental status changes	-	5	8.335	0.005
Type 2 diabetes mellitus	-	1.61	0.7008	0.405
Hypertension	-	1.14	0.06864	0.794
Congestive heart failure	-	1.11	0.01686	0.897
Coronary artery disease	-	2.13	1.260	0.265
End-stage renal disease	-	0.31	0.9486	0.333
Chronic obstructive pulmonary disease	-	2.53	1.847	0.178
Atrial fibrillation	-	1.77	0.9931	0.322
Chronic lung disease	-	1.78	0.5918	0.444
Smoker	-	1.06	0.01403	0.906
Alcoholic	-	0.71	0.3356	0.564
Rapid response team activation	-	2.53	1.847	0.178

The SIRS criteria that triggered the code sepsis did not show any pattern to suggest the predictability of ICU admission. Tachypnea (respiratory rate of >20/min) was associated with higher chances of ICU admission (OR=3.69; p=0.032; p<0.05). The patients who received the full 30 cc/kg fluid bolus had a 12.8 times higher incidence of requiring ICU admission (p=0.001; p<0.01). Hypotension in the first hour after the event had a 7.94 times higher chance of requiring ICU admission (p<0.01). Those patients met one of the quick sequential organ failure assessment (qSOFA) criteria, like hypotension with SBP <100 mental status changes, or had significantly high odds of ICU admission (OR=4.4 and 5, p<0.01). Escalation of antibiotics during the code sepsis had a 19.33 times higher chance of requiring ICU admission (p=0.002). White cell count, blood glucose level, serum creatinine, and troponins at admission were comparable in both groups. There was no statistically significant difference in the distribution of co-morbidities like type 2 diabetes, hypertension, cardiovascular or kidney disease among the patients that went to the ICU versus those that were treated on medical floors.

## Discussion

Sepsis is the systemic inflammatory response to infection characterized by a constellation of several hemodynamic, metabolic, and inflammatory phenomena. Severe sepsis is characterized by the presence of sepsis and at least one organ dysfunction (e.g., renal failure, hypoxemia, lactic acidosis, central nervous system dysfunction, liver failure, and coagulation abnormalities) [1,3]. Our study utilized a hospital-based alert system to identify patients who meet the criteria for sepsis based on SIRS criteria. Such triggers prompt the clinician to evaluate the patient at the bedside and determine if the code sepsis should be activated or canceled. Thereafter, all patients with activated code sepsis were included in the study. The study demonstrated that patients with elevated levels of lactic acid and liver enzymes, particularly AST and ALT, have a higher likelihood of being transferred to the ICU for closer monitoring. This does not necessarily mean that all patients with such laboratory values will absolutely need ICU level care, as several factors including the overall clinical picture and response of the patient to septic management are quite subjective.

Lactic acid is a byproduct of anaerobic cellular metabolism that occurs when the mitochondrial oxidative phosphorylation process is disrupted due to tissue hypoperfusion. Research has shown that elevated lactate levels increase mortality risk by 1.7 times within 90 days [3,9]. Mikkelsen et al. also observed that intermediate (2 mmol/L-3.9 mmol/L) and high lactate levels (>4 mmol/L) are associated with 22.9% of mortality at 28 days both in septic shock and non-shock groups [10]. In a study conducted by Oedorf et al., it was found that the serum lactate level had a strong correlation with adverse outcomes such as intubation, renal dysfunction, vasopressor usage, or death [11]. The correlation was observed in both infected (OR=1.94; 95% CI=1.4-2.3) and non-infected groups (OR=1.48; 95% CI=1.14-1.91). Another study showed that lactic acid enhances the predictability of a modified early warning score (MEWS), which includes variables of blood pressure, pulse, respiratory rate, temperature, and mental status [12]. The MEWS score is used to determine whether patients should be transferred to the ICU for acute care, which is consistent with the result of our study [12]. Similarly, liver failure can also result from hypoperfusion and infection, leading to liver enzymes spilling into the bloodstream. One study observed elevated AST/ALT ratio to be associated with 53% of all-cause mortality. It was a reliable diagnostic tool for diagnosing septic shock, requiring ICU transfer for vasopressor support, as depicted by our results [13]. In another study, Kobashi et al. investigated approximately 450 patients and found that septic patients with shock liver, without jaundice and cholestasis, and those with a hepatocellular pattern of liver injury with jaundice, are associated with poor outcomes [14]. However, bilirubin and ALP levels were not found to be statistically significant predictors of poor outcomes. Our study did not account for the GGT level.

Numerous studies have analyzed the effectiveness of q-SOFA, SIRS, and q-SOFA+lactate in detecting and predicting sepsis. However, our study has focused on individual components that can anticipate the outcome of septic patients. With regards to vital signs, our observations suggest that tachypnea (respiratory rate >20) is significantly associated with higher chances of admission to the ICU (OR=3.69; p=0.032). Tachypnea is a result of the patient's physiological attempt to blow off carbon dioxide to compensate for acidemia build-up due to anaerobic metabolism in critically ill patients. However, as the critical condition persists, the patient's respiratory drive may become exhausted to compensate, leading to hypercarboxemia. In such cases, intubation may be required.

According to a study conducted by Boema et al., it was found that 62.5% of critically ill patients admitted to the ICU suffer from severe respiratory failure and tachypnea. However, the study also mentioned significant missing data, which makes it essential to note that respiratory rate is often inaccurately recorded by both electronic devices and nurse's measurements [15]. Subbe et al. found that respiratory rate changes were more significant in critical patients compared to changes in heart rate or systolic blood pressure, making respiratory rate a better indicator of patient stability [16]. In terms of blood pressure, if the systolic blood pressure is below 100, the patient is at a higher risk for poor outcomes, with an OR of 4.4. Additionally, if 30 cc/kg of intravenous fluids are administered and the patient experiences persistent hypotension, the OR for poor outcomes increases to 12.8 and 7.94, respectively. In such cases, the patient may require critical care. Early goal-directed therapy, introduced over two decades ago, focuses on the early resuscitation of fluids within the first six hours in patients with severe sepsis and septic shock. It has been associated with benefits in mortality [17]. Hani et al. observed that non-compliance to delivery of guideline-directed 30 cc/kg of fluid bolus within the first three hours of sepsis identification is associated with increased odds of mortality (OR=1.52; CI=1.03-2.24), delayed hypotension (OR=1.42; CI=1.02-1.99), and increased ICU stay (~2 d) (β=2.0; CI=0.5-3.6) [18]. One of the reasons for finding administration of 30 cc/kg fluid and persistent hypotension as a statistically strong predictor of transfer to ICU could be non-compliance to sepsis bundles in the targeted time frame of three hours, which was not accounted for in our study. In addition to the six initial steps recommended by the Surviving Sepsis Organization, which include administering fluids and antibiotics, sepsis management also involves providing supplemental oxygen therapy and monitoring urine output accurately [19]. These measures can also help to redirect decisions from further fluid challenges to vasopressors in case oxygen demand increases (emerging pulmonary edema) or urine output decreases (possible congestive nephropathy).

Early administration of broad-spectrum antibiotics is a fundamental component of sepsis management. Studies indicate that patients who were escalated with antibiotics when the sepsis bundle was triggered had a 20-fold higher risk of requiring ICU level care. This could be due to a delay in initiating antibiotics within the first hour of sepsis trigger or inappropriate initial antibiotic coverage. A retrospective study by Kumar et al. reported that out of 2154 patients who received effective antibiotic therapy, those who received antibiotics within the first golden hour of persistent or recurrent hypotension had a survival rate of 80% [20]. However, for each hour of delay during the subsequent six hours, the chances of survival decreased by 7.6%. According to a study by Daniel BS et al, delaying the administration of the second dose of antibiotics after the first dose can lead to poor prognosis, including an increased risk of hospital mortality (OR=1.61; CI=1.01-2.57) and mechanical ventilation (OR=2.44; CI=1.27-4.69) [21].

Furthermore, sepsis-associated encephalopathy (SAE) is a range of cognitive dysfunctions that can cause confusion, disorientation, agitation, hypersomnolence, and even coma. The underlying causes of SAE are not yet fully understood. However, some theories suggest it may be related to oxidative phosphorylation and endothelial dysfunction, disturbances in neurotransmission, or derangements in calcium homeostasis in the brain tissue [22]. Other causative factors independently associated with SAE include hypoglycemia, hyperglycemia, hypercapnia, hypernatremia, and acute renal failure. Romanian et al. found that SAE is also associated with increased mortality, more extended hospital stays, and increased use of ICU resources [23].

Despite all these factors that can point toward critical level of care, admission to ICU does not have set criteria. It is mostly depended on hemodynamic instability of the patient that is unmanageable on medical floors and unresponsive to fluids, availability of step down units in ICU where borderline "sick" patients can be transferred, availability of resources in terms of nursing support and intensivist's decision for admission. 

Limitation

Our study has a few limitations that should be noted. First, it was conducted in a single center and was limited by the fact that certain parameters of sepsis patients might not have been detected, which could have been associated with poor outcomes requiring critical care. Second, although we do know that sepsis bundles were activated and orders were placed within the EMR during the first hour of sepsis trigger, we cannot confirm whether fluids and antibiotics were physically administered within the first hour due to retrospective data collection. Third, we were unable to establish an association between a higher risk of ICU admission after 30 cc/kg of intravenous fluid administration and cardiac and pulmonary comorbidities, possibly due to a small sample size. Due to the small size, statistical power is low and there is a hindrance in extrapolation of the result to the general population. Fourth, due to the retrospective nature and small scale of the study, we could not account for other confounding factors related to clinical decisions regarding the management and transfer of septic patients to the ICU, including the physician's expertise, nursing and ancillary staff resources, and the family's decision against vasopressor support. Last, we could not account for the association of urine output in septic patients with a higher risk of ICU transfer due to inaccurate documentation of urine output measurement on the general medical floors.

## Conclusions

In summary, it is crucial to quickly recognize and manage sepsis within the first hour of the sepsis bundle trigger to increase the chances of survival. Physicians, especially residents in training, should be trained to identify "alarming signs" in septic patients, such as tachypnea. It is important to note that the use of administrative codes (such as "code sepsis") to identify sepsis may introduce misclassification bias, with the possibility of mischaracterizing patients who do not meet the Sepsis-3 definition of "sepsis" and "septic shock." Regardless, these system-based alerts help in triaging "sicker" patients in hospitals with increased patient load per physician. Physicians should also know when to consult an ICU team if high-risk factors are observed, such as lactic acidosis, transaminitis, escalation of antibiotics, persistent hypotension, and encephalopathy. Further research is needed to identify more risk factors during the critical early hours of sepsis management to improve mortality rates and optimize ICU resources.
